# Genetic control of flowering in greater yam (*Dioscorea alata* L.)

**DOI:** 10.1186/s12870-021-02941-7

**Published:** 2021-04-01

**Authors:** Fabien Cormier, Guillaume Martin, Hélène Vignes, Laurie Lachman, Denis Cornet, Yoana Faure, Erick Maledon, Pierre Mournet, Gemma Arnau, Hâna Chaïr

**Affiliations:** 1 CIRAD, UMR AGAP Institut, 97170 Petit-Bourg, Guadeloupe France; 2 UMR AGAP Institut, Univ Montpellier, CIRAD, INRAE, Institut Agro, F-34398 Montpellier, France; 3 CIRAD, UMR AGAP Institut, F-34398 Montpellier, France; 4 Univ. Des Antilles, Pôle Guadeloupe, IUT Saint Claude, Guadeloupe, France; 5 INRA, UR ASTRO Agrosytèmes Tropicaux, Petit-Bourg, Guadeloupe France

**Keywords:** Dioecy, *Dioscorea alata*, Sex, Flowering, Breeding, GWAS, Reproduction, Yam

## Abstract

**Background:**

Greater yam (*Dioscorea alata* L.) is a major tropical and subtropical staple crop cultivated for its starchy tubers. Breeding of this dioecious species is hampered by its erratic flowering, yet little is currently known on the genetic determinism of its sexual reproduction.

**Result:**

Here we used a genome-wide association approach and identified a major genetic barrier to reproduction in yam on chromosome 1, as represented by two candidate genes. A deleterious effect on male fitness could be hypothesized considering the involvement of these two genes in male reproduction and the low frequency of this non-flowering dominant allele within the male genepool. We also extended the hypothesis of a XX/XY sex-determination system located on chromosome 6 in *D. alata* to encompass most of the species diversity. Moreover, a kompetitive allele-specific PCR (KASPar) marker was designed and validated that enables accurate cultivar sex estimation. The reconstruction of chromosome 6 associated with the detection of highly putative structural variations confirmed the possible involvement of a major part of the chromosome.

**Conclusion:**

The findings of this study, combined with proper estimation of accession ploidy levels to avoid endosperm incompatibility issues, could facilitate the design of future promising parental combinations in *D. alata* breeding programs. Moreover, the discovery of this genetic barrier to reproduction opens new avenues for gaining insight into yam reproductive biology and diversification.

**Supplementary Information:**

The online version contains supplementary material available at 10.1186/s12870-021-02941-7.

## Background

Conventional plant breeding programs have to generate large numbers of progenies to increase the chance of selecting new varieties with the desired phenotypes. The development of high-throughput phenotyping and genotyping methods has markedly enhanced the prediction of better parental combinations. An effective strategy could be to focus on few selected parental combinations and generate large populations [1]. However, the parental crossing ability (e.g. fertility, synchronization, compatibility) could still be a major issue. Indeed, self-incompatibility is a common barrier among angiosperms [2]. Moreover, multiple interacting exogenous and endogenous signals are involved in flowering [3]. Hence, knowledge and control of the plant reproductive biology is essential for efficient plant breeding and substantial scientific research has been devoted to this.

In root and tuber crops, the reduced ability of sexual propagation is directly inherited from domestication and diversification processes [4]. Traits related to sexual reproduction are no longer highly maintained or directly counter-selected due to the associated costs. For example, in potato (*Solanum tuberosum* L.), comparative genomic studies between wild and cultivated forms have revealed a selection signature on genes involved in pollen development and gametogenesis [5]*.* In addition, extensive clonal propagation of some cultivars can also disrupt the functioning of sexual systems [6]. More generally, ploidy levels and dioecy are also direct barriers to sexual reproduction, yet in this sense edible yams cultivated for their starchy tubers and whose dioecy is a key character [7] are not an exception. This is especially the case for greater yam (*D. alata*), which is a polyploid species [8] with no known ongoing gene flow with its wild relatives [9].

Greater yam is the most widespread yam species [10]. Despite its cultural, economic and nutritional importance, cultivated varieties are mostly landraces (e.g. in Côte d’Ivoire [11]) as breeding programs are struggling with the relatively low crossing success. Incompatibility/sterility issues due to ploidy levels [10] were overcome once the basic chromosome number (2n = 40) was confirmed [8, 12] and the failure of crosses, due to the use of triploid parents (2n = 60) or endosperm incompatibility, was understood [13]. Polyploid accessions have been successfully used and/or created [12–14], but the success of crosses has still not been explained. Moreover, significant differences have been revealed in seed sets obtained between parental combinations, in addition to ploidy issues [14, 15]. Segregation distortion in biparental populations also suggests that gametophyte and/or zygotic selection may occur [16].

Breeding programs are mainly hampered by the erratic and asynchronic flowering of *D. alata* [17], thus limiting the number of compatible fertile parents and consequently the number of successful crosses. Most studies carried out so far to understand the yam reproduction biology have focused on sex-determination in narrow intra-species diversity circumstances. Indeed, ZW/ZZ (*D. rotundata* [18]) and XX/XY (*D. floribunda*, [19]; *D. tokoro*, [20];; *D. alata*, [16]) sex-determination systems have been described using only biparental populations. Moreover, although the assumption of sterility related to polyploidy has been disproven, as previously mentioned [14], the possibility of female sterility is a relevant conjecture as most flowering accessions are males (*D. alata*: [14]; *D. rotundata*: [21]). Further studies are needed on a more diverse range of yam accessions to be able to draw conclusions on these findings.

The aim of this study was to gather further knowledge on yam reproductive biology to strengthen breeding programs. We thus focused on *D. alata*, to: (i) identify the presence of any genetic barriers to flowering in *D. alata*, and (ii) extend current knowledge on sex determination to broader and more diverse range of yam varieties.

## Results

### Panel descriptions and phenotype distributions

Two panels were used to perform the genome-wide association studies. The panel used to study flowering ability consisted of the 122 accessions: 88 assessed as being flowering forms (40 females and 48 males) and 34 as being non-flowering forms. The panel used to identify sex determinism consisted of the 88 accessions in the previous panel of known sex plus two more accessions (41 females and 49 males). The structure was significant in both panels, and the first PCA axis explained more than 40% of the variance (Additional file 1: Fig. S1; Additional file 2: Fig. S2). This was mostly due to the presence of a group of triploid female accessions that clustered apart.

We also noticed that two male genepools were present. One consisted of accessions integrated in the CRB-PT collections from the INRAe breeding program in Guadeloupe, along with ‘Pyramide’, one of their genitors. The other consisted of accessions belonging to the clonal lineage of the ‘Kabusa’ landrace. Male and female accessions were homogeneously distributed in other genepools. Concerning the flowering ability, a more homogeneous distribution of phenotypes within the range of diversity was observed.

For quantitative trait nucleotide (QTN) detection, the moderate to null increase in the observed *p*-values compared to the expected p-values, as revealed by Q-Q plot analysis, confirmed that the panel structure and kinship were well controlled using the (P + K) GWAS (Genome Wide Association Studies) model (Additional file 1: Fig. S1; Additional file 2: Fig. S2).

### Genetic control of flowering ability

**Fig. 1:**
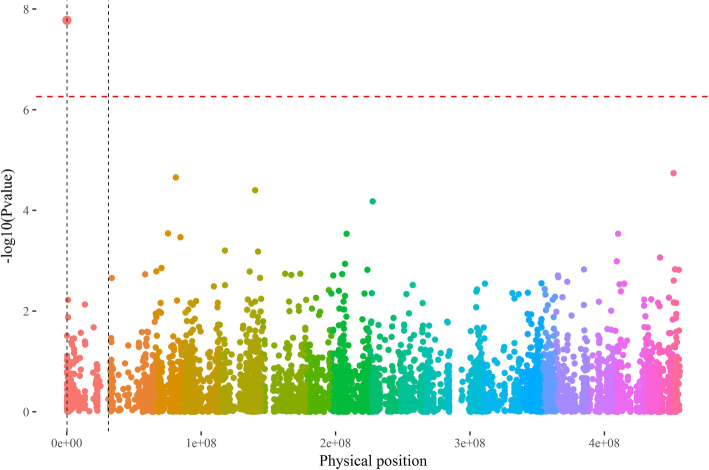
Manhattan plot of GWAS (P + K model) conducted on flowering*. Color, D. rotundata pseudo-chromosome v1* [18]*; red dashed horizontal lines, FDR threshold at a risk of 1%; dashed black vertical lines = chromosome 1 boundaries*

GWAS was first conducted to identify QTN related to flowering phenotypes. Only one QTN was detected (01.1_172298); when using the (P + K) model at a false discovery rate (FDR) risk of 1% (Fig. 1). This locus was located on pseudo-chromosome 1 at the 172,798 bp position in the *D. rotundata* genome v1 [18], corresponding to scaffold112 of the *D. alata* genome v1 at the 17,773 bp position (Water Yam Genome Project – ftp://yambase.org).

At this locus, only two genotypes were observed through GBS, i.e. homozygous CC and heterozygous CT, with the T allele associated with non-flowering phenotypes. Indeed, 89.7% (26/29) of the genotyped accessions assessed as non-flowering were heterozygous CT and 93.7% (45/48) of the male accessions were homozygous CC. However, female accessions were genotyped as either CC or CT in a similar proportion (Table 1).

**Table 1:** Contingency table of phenotypes and genotypes, assessed by GBS, at the sex-related (06.1_19660282) and the flowering-related (01.1_172298) loci

Sex	06.1_19660282	01.1_172298			
		C/C	C/T	NA	Total
Female	A/A	**18**	**14**(1)	2	**35**
	A/G	1	3	–	4
	NA	–	2	–	2
Male	A/A	4	(1)	–	5
	A/G	**38**	2	–	**40**
	G/G	–	1	–	1
	NA	3	–	–	3
Non-Flowering	A/A	3	**19**	3	25
	A/G	–	5	2	7
	NA	–	2	–	2
Total		67	50	7	124

Accessions that were only used in the GWAS for sex determination and not for flowering ability are shown in brackets. *NA*; missing data

Only the beginning of scaffold112 of the *D. alata* genome v1 was actually mapped on the draft *D. alata* chromosome 1 (see in [16]) and the sequence homology between *D. alata* and *D. rotundata* was good (Additional file 3: Fig. S3A). *D. rotundata* was thus used as a reference upon which the *D. alata* transcriptome was aligned in order to avoid issues related to *D. alata* scaffolding and to extend our research of candidate genes prior to the beginning of scaffold112.

Gene ontology analysis revealed that the genomic region encompassing the QTN related to flowering ability was significantly enriched in genes involved in the reproduction process and gamete generation (Additional file 3: Fig. S3B). This was due to the presence of two genes expressed in *D. alata*: the first one at around 125 kb (*D. alata* transcriptome contig7439) annotated as a cyclin-dependent kinase F-4 CDKF4 and the second around 167 kb (*D. alata* transcriptome contig699) annotated as an E3 ubiquitin-protein ligase SINAT2.

### Detection of sex-determination loci

**Fig. 2:**
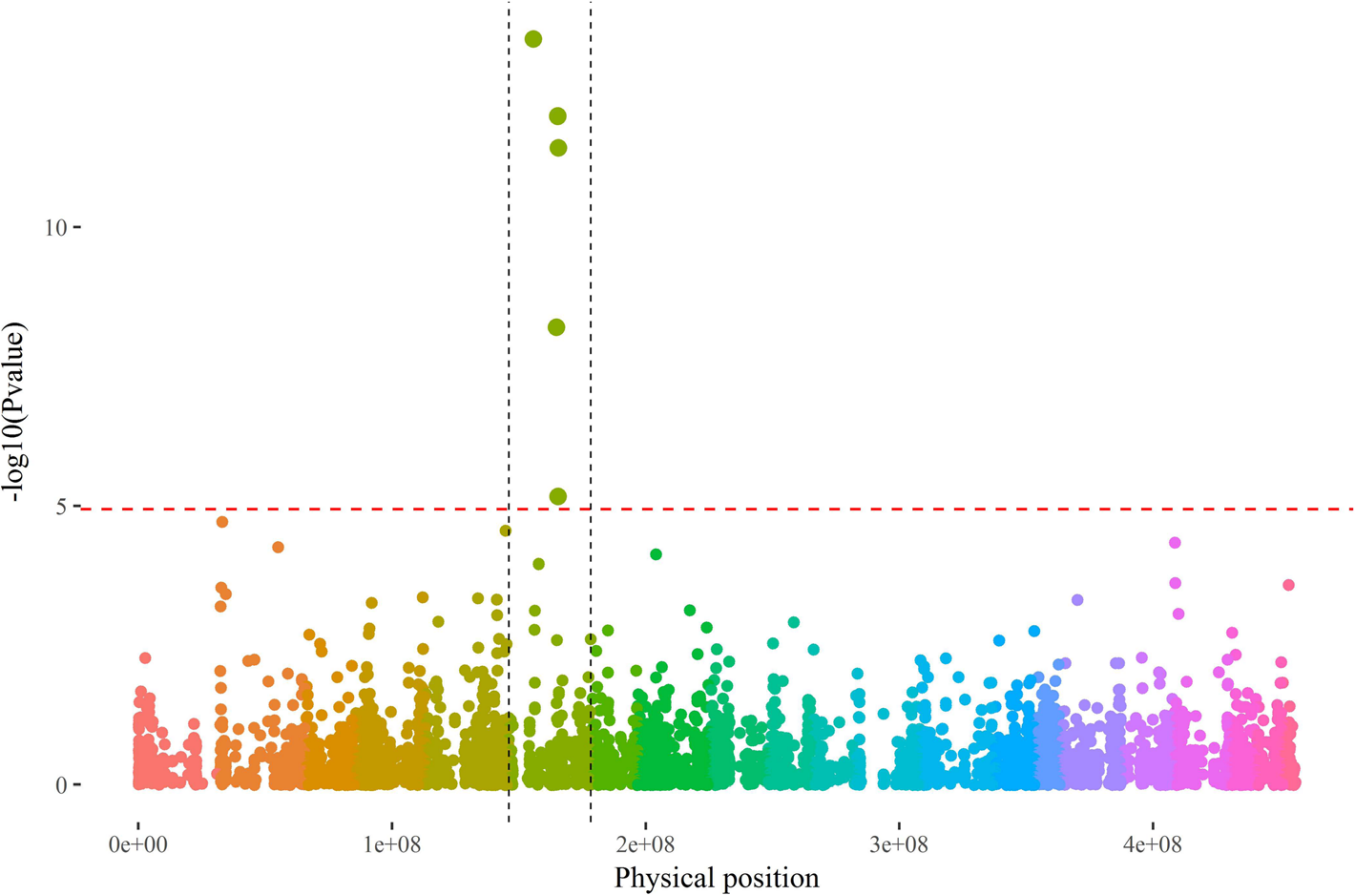
Manhattan plot of GWAS (P + K model) conducted on sex determination. *Color, D. rotundata pseudo-chromosome v1 (Tamiru* et al.*, 2017); red dashed horizontal lines, FDR threshold at a risk of 1%; dashed black vertical lines = chromosome 6 boundaries*

GWAS was also conducted using flowering accessions to identify sex-related loci. Using an FDR threshold of 1% and a (P + K) model, significant sex-linked QTNs were only located on chromosome 6 (Fig. 2). Those five QTNs were positioned from 9,886,520 to 19,660,282 bp on the *D. rotundata* pseudo-chromosome 6 v1 and their -log10(*p*-value) ranged from 5.7 to 13.37.

At the most predictive QTN, the allelic composition assessed by GBS was in agreement with the observed sex for 89% of the accessions (76/85; Table 1). This locus was positioned at 19,660,282 bp, with females being mostly homozygous (A/A) and males mostly heterozygous (A/G), or homozygous (G/G) for one accession.

Flanking sequences of the corresponding Single Nucleotide Polymorphism, SNP (06.1_19660282) were extracted to design allele-specific KASPar primers (X = A; Y = G). Then this KASPar assay was validated on 42 different accessions (Table 2; Additional file 4: Fig. S4). All tetraploid males were genotyped as XXXY, indicating that their allelic composition was AAAG. Diploid males were genotyped as XY (AG) and diploid females as XX (AA). The only two exceptions were: a XXXY (AAAG) tetraploid female (‘Noulelecae’) and a XX (AA) diploid male (‘Peter’).

**Table 2:** Results of KASPar genotyping of 42 accessions regarding their ploidy and sex

Sex	Ploidy	KASPar genotyping				
		XX	XXX	XXXX	XY	XXXY
**F**	**2**	7				
	**3**		8			
	**4**			3		**1**
**M**	**2**	**1**			16	
	**4**					6

*The KASPar assay was designed to amplify allele-specific sequences at the sex-determination locus 06.1_19660282*

To conclude, the efficiency of this KASPar assay as a diagnostic tool for sex determination was thus estimated at 95% (40/42). Moreover, the hypothesis of the presence of a dominant Y allele and a recessive X allele, in agreement with the XX/XY sex-determination system, appeared to be validated.

### Chromosome reconstruction

**Fig. 3:**
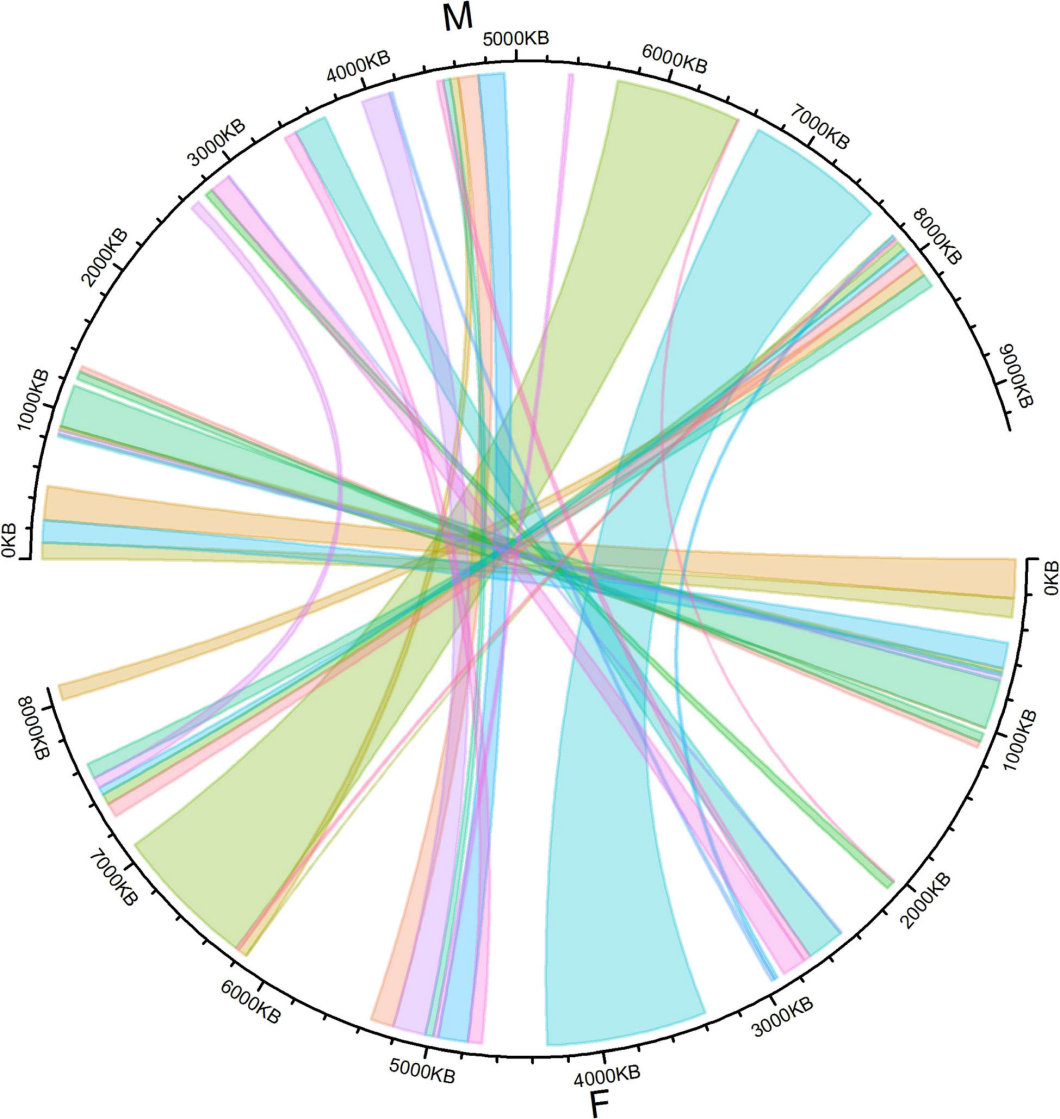
Circos visualization of sequence homology between the male and female reconstructed *D. alata* chromosome 6

A biparental segregating population was used to reconstruct a *D. alata* male and a female chromosome 6 from the available unordered scaffolds. On the male side, 240 high quality segregating SNPs were identified as belonging to linkage group 6, including 223 that were positioned with confidence on a total of 76 *D. alata* scaffolds v1. On the female side, 164 high quality segregating SNPs were identified as belonging to linkage group 6, including 154 that were positioned with confidence on a total of 61 *D. alata* scaffolds v1. Twenty positioned SNPs and 35 scaffolds were in common between both parents. Scaffolds were then ordered and oriented per parent using pairwise recombination frequencies between the positioned SNPs. One to 36 SNPs per scaffold (mean 2.9) were then used for males, while one to 11 SNPs per scaffold (mean 2.5) were used for females. Finally, the reconstructed male and female genomic sequences corresponding to chromosome 6 had a total length of 9,306,440 bp and 8,100,612 bp, respectively, with a cumulated length of 4,392,624 bp in common (Fig. 3).

**Fig. 4:**
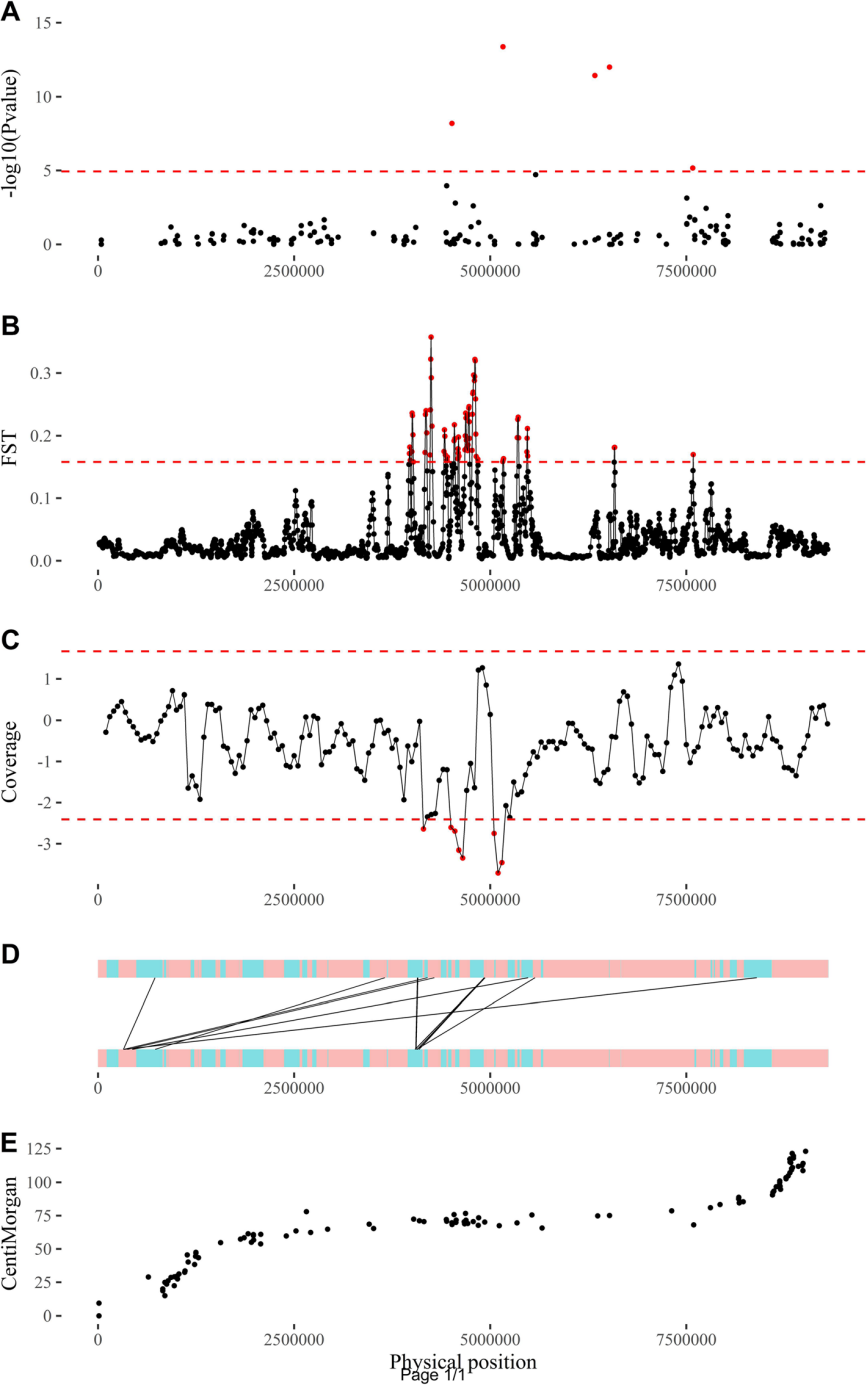
**a**) GWAS on sex-determination results, **b**) mean FST on an SNP sliding window along the chromosome (step = 100, window = 500), **c**) mean coverage difference between male and female pools on a physical (bp) sliding window (step = 50,000, window = 200,000 bp), **d**) male-specific discordant read clusters, and **e**) physical versus genetic position on the reconstructed male chromosome 6*. Red horizontal dashed lines, significance threshold at a risk of 0.01: (A) FDR at 1%, B) and C) using a gamma distribution fitted on empirical value distributions; red points, significant positions*

Both chromosomes were reconstructed with confidence in the light of the pairwise recombination frequencies (Additional file 5: Fig. S5) and the accurate correspondence between the physical and genetic distances (Additional file 6: Fig. S6). The GWAS sex determination results were therefore plotted according to the SNP position on the reconstructed male chromosome 6. This revealed that the genomic region related to sex spanned more than 3 Mb from 4.51 Mb to 7.58 Mb if significant SNPs were taken as borders (Fig. 4a).

### Selection signature and structural variation

Several approaches were implemented to refine the location of the sex-related region and chromosome structure. Two male and two female DNA pools from biparental populations were first resequenced. This generated a total of 710 million paired-end reads, 6.09% of which were mapped on the reconstructed male chromosome 6 and used thereafter.

From those reads, SNP detection and filtering procedures identified 188,947 high-quality polymorphic sites within the four DNA pools. Highly significant differentiation (*Fst*) between males and females were detected and located within the centromeric region of the male chromosome 6 (Fig. 4b; Additional file 7: Fig. S7). Moreover, this result was closely in line with previous results obtained on the diversity panel (Fig. 4a).

Mapped reads were then pooled by sex to study the read coverage and perform structural variation detection. The median read coverage along this chromosome was assessed as being 19 and 20 for male and female pools, respectively. Regarding the window size used to assess differences in read coverage, the first results showed that a large region was significantly less covered by male reads than by female reads (Fig. 4c; Additional file 6: Fig. S6) around 5 Mb.

At a finer scale, a total of 59 female and 66 male discordant read clusters (probably related to structural variations) were detected, nine of which were female specific and 16 were male specific. Male specific discordant read clusters mostly converged within the already highlighted centromeric region (Fig. 4d; Additional file 8: Fig. S8).

## Discussion

### Flowering ability

As mentioned in the introduction, *D. alata* breeding programs are hampered by the erratic flowering pattern of this species, thus reducing its crossing potential. This means that most accessions do not flower yearly, and the flowers are sparse once they do flower [22]. Phenotypes should thus be monitored in several conditions in order to differentiate “real” non-flowering accessions from environmentally-dependent flowering accessions, hence facilitating accurate estimation of the genetic value and GWAS. We assumed that non-flowering phenotypes may have been properly assessed via recurrent agro-morphological characterization of the CRB-PT collection. Here we documented a plausible genetic barrier to reproduction in yam for the first time by highlighting a dominant allele related to non-flowering phenotypes in *D. alata*.

Evidence of the involvement of the genomic region in the reproduction process (i.e. male gamete generation) was strengthened via the presence of two candidate genes: one homologous to CDKF4 of *Oryza sativa* L. and another to SINAT2 of *Arabidopsis thaliana*. CDKF4 belongs to a large family of serine/threonine protein kinases conserved among eukaryotes and involved in cell cycle regulation [23]. It was found to be highly expressed in *O. sativa* L. flower buds (NCBI BioProject: PRJNA243371), especially in mature pollen ([24], as well as in cotton petals and stamens [25]. Moreover, SINAT2 belongs to E3 ubiquitin-ligases, which are known to be key phytohormone signalling regulators [26]. In *A. thaliana*, it is highly and mostly expressed during pollen development (The Arabidopsis Information Resource [27];). Its activity is modulated by CDKG1 [28], which is involved in thermal-sensitive male meiosis [29].

Less specifically, both genes were also found to be involved in abiotic stress responses (e.g. [25, 30]). Moreover, the generation of male gametes and/or the effect on male meiosis does not seem to be directly related to the plant flowering ability, which could presumably be more related to mechanisms such as floral organ genesis. However, as ubiquitin-mediated control and serine/threonine kinases are complex central networks in plants, further studies focused on yam would be warranted to gain insight into their possible involvement in yam flowering.

The non-flowering dominant allele was also present in half of the female accessions and almost absent in the male accessions, so it was in high linkage disequilibrium (LD) with the sex-related locus, but not on the same chromosome. The fact that long-range LD maintenance can be promoted by selection [31] suggests that this allele has a deleterious effect on male fitness. Indeed, if it actually reduces/annihilates male fitness, selection likely occurred and reduced its frequency within the male genepool.

### Sex determination and sex chromosomes

The presence of an XX/XY sex-determination system located on chromosome 6 of *D. alata* was first identified using the quantitative trait loci QTL approach in two biparental populations [16]. However, that finding could have been related to the parental specificity, especially to the single female used to generate those two populations. In this study, we confirmed both the presence of an XX/XY sex-determination system and the location of the genomic region involved. Furthermore, we extended those findings to a more diverse range of *D. alata* accessions using GWAS.

Two hypotheses could explain the large size of the sex-related QTL detected in [16], i.e. the small populations size and/or the presence of a low recombinant region related to heteromorphic sex chromosomes [32]. Here our results confirmed that a large centromeric region was involved in sex determination in *D. alata*. Significant differences between the male and female chromosome 6 were also highlighted. In addition, highly putative male specific structural variations were detected between the sequenced male pools and the reconstructed male chromosome 6, thus supporting the heteromorphic chromosome hypothesis.

The reconstructed male chromosome 6 was actually biased. The classical S shape noted between the genetic and physical distance suggested that SNPs and thus scaffolds were ordered with confidence. However, SNPs were detected so their corresponding genomic regions were common to the male and female parents. The scaffolds used were also generated from the female accession Tda9500038 (*D alata* v1; Water Yam Genome Project – ftp://yambase.org). If Y-specific sequences actually existed they were not present within the assembly we designed, so the male chromosome generated would more likely have been a consensus between the putative Y and X chromosomes since X and Y chromosomes conserve homolog sequences by meiotic pairing and exchange [33]. Moreover, it could be hypothesized that the significant coverage difference between male and female reads within the sex-related region was due to the presence of a single X copy in males (XY) and the two X copies in females (XX).

### Implications for yam breeding

The selection of somaclonal mutants generated the current *D. alata* cultivated diversity ([9, 34]. Male and female phenotypes, as well as flowering ability, were spread throughout the studied diversity panel (Additional file 1: Fig. S1; Additional file 2: Fig. S2). Both phenotypes may thus not have arisen via mutations in specific gene pools. However, they may have appeared early during the species diversification process, suggesting that all gene pools could theoretically be used in conventional breeding programs.

The highly qualitative phenotypes used in this study, i.e. sex and flowering ability, could have been assessed on the basis of a single observation over the several years of morphological characterization within the CRB-PT collections (i.e. if the accession only flowered once). Indeed, high variations among accessions on flowering abundance/frequency and pollen viability have been reported in yam (e.g. [14]). Other minor locus effects that could possibly explain the significant proportion of flowering ability variance should nevertheless now be investigated. For example, the ‘Peter’ male accession genotyped as a female (XX) is known to produce a few small flowers with a low pollen viability rate (E. Maledon, pers. comm.).

The ploidy levels of accessions could also possibly explain the mismatch between the genetic factors highlighted in this study and phenotypes. Indeed, polyploidy leads to major changes in gene regulation and expression [35]. For example, the only female accession genotyped as having a Y allele was Noulelcae, i.e. a tetraploid. Interestingly, among the 19 female accessions genotyped as having the non-flowering allele, 13 were polyploids (four tetraploids and nine triploids).

Sex and non-flowering phenotypes nevertheless seem to be mainly genetically determined, so our identified markers could thus be effectively used for sex and non-flowering prediction. Early accurate estimation of possible parental combinations could now be conducted in breeding programs prior to designing crosses. Such initiatives could be combined with sex/flowering genetic determination and ploidy level assessment, to avoid endosperm incompatibility. Moreover, pinpointing the genetic factor controlling the flowering ability could help identify the environmental conditions conducive to flowering (e.g. genotype-dependent temperature or day length responses), while facilitating the development of protocols to promote flowering induction as is currently the case regarding cassava flowering [36] and yam tuber dormancy [37].

## Conclusions

Genome-wide association studies were performed to study greater yam (*D. alata*) flowering ability and sex determination. The study findings highlighted a genetic contribution to flowering ability located on chromosome 1 and the expression of two genes, one homologous to CDKF4 of *Oryza sativa* L. and another homologous to SINAT2 of *A. thaliana.* Moreover, we confirmed that a dominant male-related allele was present on chromosome 6 and that a large portion of the chromosome was involved, thereby supporting the hypothesis of an XX/XY sex-determination system. We thus designed a KASPar assay as a diagnostic tool for sex determination. These interesting findings could pave the way for identifying future parental combinations, while facilitating breeding for traits of interest such as tuber quality and disease resistance. They should also help gain further insight into this crop diversification process.

## Methods

### Plant materials

A total of 124 yam (*D. alata*) accessions maintained in the West French Indies (Guadeloupe) at the Tropical Plants Biological Resources Centre (CRB-PT) and CIRAD were used for GWAs analysis in order to identify regions related to sex determination and flowering capacity (Additional file 9: Table S1). Sex was determined by mining CRB-PT agro-morphological description data (available at http://intertrop.antilles.inra.fr) and by assessing CIRAD accessions during the flowering period (December to January). Female and male phenotypes were coded as 0 and 1, respectively, for a total of 90 accessions. Regarding non-flowering phenotypes, accessions were assessed as non-flowering forms if they had been morphologically characterized by CRB-PT, however no information had ever been recorded regarding their sex. Flowering (male or female) and non-flowering phenotypes were coded as 0 and 1, respectively, for a total of 122 accessions, including 88 out of the 90 sex-determined accessions.

Progenies of two F1 outcrossed populations involving one female (74F) and two males (Kabusa and 14 M), described in [16] as populations A (74F × Kabusa) and B (74F × 14 M), were also used. Both populations were used to detect the selection signature and structural variations on the male sex chromosome. Population A was also used to create sex-chromosome reference sequences.

### Genotyping-by-sequencing and SNP filtering

Exactly the same DNA extraction, genotyping-by-sequencing (GBS) and SNP calling and prefiltering protocols described in [16] were applied. They were based on the DNA extraction procedure described in [38], the genotyping by sequencing described in [39] and the SNP calling and prefiltering using process reseq.1.0.py software and VcfPreFilter.1.0.py implemented in the VcfHunter package [40]. Raw sequencing reads were obtained from [9] and aligned on the *D. rotundata* reference genome v1 (pseudo chromosome BDMI0100001–21 [18]; to detect SNPs. This procedure was applied to the two panels used in GWAS and to the biparental population (population A, 74F × Kabusa) used to reconstruct sex chromosomes.

Concerning the datasets from the pre-filtered vcf files used in GWAS, SNPs and accessions were filtered using the following filters: minimum depth 8, minor allele frequencies per site > 5%, maximum missing data per loci < 10% and maximum missing data per accession < 20%. To generate the genotyping incidence matrices, genotypes homozygous for the reference allele, heterozygous or homozygous for the alternate allele were respectively converted to 0, 1 and 2 regardless of the accession ploidy level. Finally, matrices consisting of 90 accessions × 4973 SNPs and 122 accessions × 6033 SNPs were used to perform GWAS for sex and flowering ability, respectively.

To reconstruct the sex chromosomes (population A), SNPs and progenies were filtered from the pre-filtered vcf files based on the following criteria: minimum depth 8, maximum missing data by SNP < 20%, maximum missing data per progeny < 50%, at least 100 bp between consecutive SNPs, adequate segregation between parents (homozygous in one parent or heterozygous in both) and a segregation distortion χ^2^ test *p*-value <1e-4. Then SNPs were assigned to male and female datasets regarding their segregation pattern within the parents. This resulted in a genotyping male matrix of 110 progenies × 5473 SNPs consisting of SNPs heterozygous in the male parent and homozygous in the female parent, or heterozygous in both parents; and a female matrix of 110 progenies × 5866 SNPs consisting of SNPs heterozygous in the female parent and homozygous in the male parent, or heterozygous in both parents. SNP and accession filtering was conducted with R scripts (R 3.4.4, R Core Team, 2017) using the vcfR 1.5.0 package [41].

### Genome-wide association studies

SNP-trait associations were first tested using a generalized linear model coded in R using the glm function. According to the [40] method, based on principal component analysis (PCA) of a modified genotyping incidence matrix, the panel structure was investigated and tested using a Tracy-Widow test. As only the largest eigenvalues were assessed as being significant (*p* < 0.001), the panel structure was estimated using accession coordinates on the first PCA axis. Then the mixed model procedures Q (i), K (ii), and Q + K (iii) developed by [42] were applied using the ASReml-R package [43] and expressed as:

$$ \mathrm{y}=1\upmu +\mathrm{Q}+\mathrm{S}\upalpha +\upvarepsilon\ \left(\mathrm{i}\right) $$

$$ \mathrm{y}=1\upmu +\mathrm{S}\upalpha +\mathrm{Zu}+\kern0.5em \upvarepsilon \kern0.5em \left(\mathrm{ii}\right) $$

$$ \mathrm{y}=1\upmu +\mathrm{Q}+\mathrm{S}\upalpha +\mathrm{Zu}+\kern0.5em \upvarepsilon \kern0.5em \left(\mathrm{iii}\right) $$

where y is a vector of phenotypes coded as 0 or 1; 1 a vector of 1; μ the intercept; Q is the vector of accession coordinates on the significant PCA axis resulting from the panel structure analysis; α is the additive effect of the tested SNP; u is a vector of random polygenic effects assumed to be normally distributed N(0,σ^2^yK); where K is a matrix of relative kinship computed as the percentage of shared alleles, S and Z are incidence matrices, and ε is a vector of residual effects.

### Validation

Regarding the results, two types of validation were performed: genotyping using KASPar technology or a gene ontology study.

SNP conversion for the KASPar assay was conducted as described in [44], except that the wet chemistry was conducted at the CIRAD Roujol research station (Guadeloupe, France). Polymorphic SNP flanking sequences (60 bp upstream and 60 bp downstream around the variant position) were selected using SNiPlay3 [45]. The LGC KASP master mix (standard protocol with 31 PCR cycles) was used once DNA had been extracted from leaf tissue using the DNeasy**®** Plant Mini Kit (standard extraction protocol, Qiagen). Overall, 42 accessions with known ploidy levels [9] were genotyped and 8 negative controls (water or water and mix) were included in the experiment.

Gene ontology (GO) enrichment was tested using the TopGO R cran package (“classic” Fisher test options). GO terms were extracted from the annotated *D. alata* transcriptome [46] mapped on the *D. rotundata* genome v1 available at http://yam-genome-hub.cirad.fr/jbrowse.

### Chromosome reconstruction

Chromosome reconstruction was based on marker segregation within the biparental population A (74F x Kabusa), consisting of 110 progenies. Linkage analysis was conducted on a per-parent basis, leading to separate reconstruction of a female and a male chromosome.

First, from the filtered genotyping matrices obtained through GBS and vcf filtering, as previously described, 20 linkage groups were defined using JoinMap 4.1 software [47], while setting the grouping LOD threshold at 7/8 for both parents. SNPs belonging to linkage group 6 (LG6 corresponding to chromosome 6) were identified and their position on the unordered scaffolds of the *D. alata* genome v1 (Water Yam Genome Project – ftp://yambase.org) was assessed using the Basic Local Alignment Search Tool (BLAST) according to the procedure described in [44].

Then *D. alata* scaffolds were ordered and oriented (when possible) based on pairwise recombination frequencies between the SNPs they contained. This was done using the Southgreen Galaxy “Chromosome reconstruction” pipeline based on the Scaffrehunter package [48]; available at http://galaxy.southgreen.fr/galaxy/u/droc/p/scaffhunter%2D%2Dchromosome-reconstruction).

Homology between the male and the female reconstructed *D. alata* chromosome 6 was then visualized using a Circos approach via the circlize R package [49].

### DNA extraction and resequencing

The differentiation and structural variation studies were based on resequencing of four pools of DNA: two pools of male or female progenies from the two biparental populations 74F × Kabusa (36 females and 38 males) and 74F × 14 M (32 females and 46 males). Each pool consisted of eight leaf punches per flowering progeny to balance the DNA quantity and by choosing leaves close to flowers to avoid vine mixing. DNA extraction and quality checks were carried out as previously described at the GenoAgap platform (CIRAD, Montpellier, France). Library preparation and sequencing was conducted using the Illumina TruSeq PCR-Free kit. Paired-end (2 × 150 bp) sequencing was conducted on an Illumina HiSeq3000 system. Both library preparation and sequencing were performed at the GeT-PlaGe platform (INRAe, Castanet-Tolosan, France).

### Detection of differentiation and structural variation

Male and female differentiation was studied using a F*st* approach based on SNPs detected among the four DNA pools on the reconstructed male chromosome 6, as described for the GBS procedure, using the following parameters: minimum depth 20, minor allele frequencies per site > 15% and no missing data. F*st* variation along the chromosome was assessed using a sliding window of 500 SNPs (step = 100 SNPs). The significance threshold was calculated at a probability of 0.01 of a gamma law fitted on the resulting F*st* distribution.

Structural variation was detected using the scaffremodler package [48] available at https://github.com/SouthGreenPlatform/scaffremodler. First, male and female resequencing datasets were generated by combining raw reads by sex. These male and female datasets were separately used on the reconstructed male chromosome 6 with the following parameters: minimum read insert size = 150, maximum read insert size = 450 and the very sensitive end-to-end mapping process of bowtie2 [50]. Then male-specific clusters of discordant reads (probably related to structural variations) were manually identified by comparing the two results files.

From the paired-end read mapping performed during the detection of clusters of discordant reads, the coverage difference between male and female pools was calculated as the mean difference between male and female sequencing depths in a sliding window of 200,000 bp (step = 50,000 bp). The significance threshold was calculated at a probability of 0.01 of a normal law fitted on the resulting difference in distribution coverage.

## Additional Files

**Additional File 1: Fig. S1.** Details on GWAS on sex determination (female or male flowering accessions). A) Barplot of the first 10 PCA eigenvalues computed to assess the panel structure. B) Clustering of accessions based on coordinates on the first five PCA axes. R cran, hclust function, “ward.D2” method. Red, female accessions; blue, male accessions. C) QQplot on GWAS results. Up/left, generalized linear model; up/right, P model; down/left, K model and down/right PK model.

**Additional File 2: Fig. S2.** Details on GWAS on non-flowering phenotypes. A) Barplot of the first 10 PCA eigenvalues computed to assess the panel structure. B) Clustering of accessions based on coordinates on the first five PCA axes. R cran, hclust function, “ward.D2” method. Purple, non-flowering accessions; black, flowering accessions. C) QQplot on GWAS results. Up/left, generalized linear model; up/right, P model; down/left, K model and down/right PK model.

**Additional File 3: Fig. S3.** Details on the genomic region linked to non-flowering/flowering phenotypes. A) Dotplot of sequencing homology between *D. rotundata* and *D. alata*. The dotplot was computed using the NCBI blastn web server (discontinuous megablast, default parameter) with the *D. rotundata* sequence of chromosome BDMI01000001.1 (*D. rotundata* genome v1 [18];) on the y-axis and the *D. alata* scaffold112 (*D. alata* genome v1) on the x-axis. B) Summary of significantly enriched G.O. terms within the genomic region. G.O. terms were extracted from the annotated *D. alata* transcriptome [46] mapped on the *D. rotundata* genome v1 and available at http://yam-genome-hub.cirad.fr/jbrowse. G.O term enrichment analysis was performed using the TopGO R cran package (“classic” Fisher test options) studying the *D. rotundata* genomic region from 50 kb to 250 kb on *D. rotundata* chromosome BDMI01000001.1.

**Additional File 4: Fig. S4.** Details on KASPar validation of the sex-related SNP (06.1_19660282). A) SNP flanking sequence and B) KASPar fluorescence results. Fluorescence signals are plotted by accession, ploidy and observed sex. In x, the “A” fluorescence allele, and in y, the “G” fluorescence allele.

**Additional File 5: Fig. S5.**: Dotplot of recombination frequencies along the reconstructed male and female chromosome 6 of *D. alata*. A) Male chromosome 6 and B) Female chromosome 6. Recombination frequencies were computed from a biparental population (74F x Kabusa) consisting of 110 progenies. Scaffolds from the *D. alata* genome V1 were used. Chromosome reconstruction pipeline available at: http://galaxy.southgreen.fr/galaxy/u/droc/p/scaffhunter%2D%2Dchromosome-reconstruction.

**Additional File 6: Fig. S6.** Physical versus genetic distance along the two reconstructed sex-chromosome 6. Up, male chromosome; down, female chromosome. Genetic distance were calculated using JoinMap 4.1 software (Van Ooijen, 2012; option: recombination frequencies below 0.45, LODs over 1.0, ripple value 1, regression mapping and Kosambi mapping function).

**Additional File 7: Fig. S7.** Details on significance thresholds used in male and female resequencing comparative studies. A) Distribution of Fst between male and female and definition of significance threshold. A sliding window of 500 SNPs was used (step = 100 SNPs) to compute Fst. The significance threshold was then assessed at 0.158. B) Difference in male and female read coverage. A sliding window of 200,000 bp was used (step = 50,000 bp). Thresholds were assessed at 6.67 and 2.59 for significance of over-coverage and under-coverage for males and females, respectively. For both analyses, a gamma distribution was fitted on the empirical distribution using the fitdist function of the fitdistrplus R cran library (red curves). The significance thresholds (vertical red lines) were then assessed using a risk of 0.01.

**Additional File 8: Fig. S8.** Circos visualization of male-specific discordant read clusters. Discordant read clusters (putative structural variations) were detected using the scaffremodler package (Martin et al., 2017) available at https://github.com/SouthGreenPlatform/scaffremodler. Male and female resequencing datasets were separately used on the reconstructed male chromosome 6. Default options were used with an expected forward-reverse read orientation and a minimal and maximal insert size set at 150 and 450 bp, respectively. Then male-specific discordant read clusters were trimmed by comparing the two results files. Links color: red, deletion; blue, reverse-forward; Scaffolds, black or yellow; Barplot from black to blue depending on the male read depth and the plot of scaled coverage difference between male and female pools (step: 500 bp; window: 1000 bp).

**Additional File 9: Table S1.** Accessions used in this study with their attributes: flowering and sex.

## Data Availability

The Illumina Hiseq 3000 sequencing raw data are available in the NCBI SRA (Sequence Read Archive), under the BioProject number: PRJNA592336 and PRJNA515897. KeyGene N.V. owns patents and patent applications protecting its Sequence Based Genotyping technologies.

## Abbreviations

CDKF4: cyclin-dependent kinase F-4
CRB-PT: Centre de Ressouces Biologique-Plantes Tropicales
DNA: DeoxyriboNucleic Acid
FDR: False discovery rate
GBS: Genotyping by sequencing
GWAS: Genome Wide Association Studies
INRAe: Institut national de la recherche agronomique
KASPar: Kompetitive allele-specific PCR markers
LD: Linkage disequilibrium
PCA: Principal component analysis
QTN: Quantitative trait nucleotide
QTL: Quantitative trait loci
SINAT2: E3 ubiquitin-protein ligase
SNP: Single Nucleotide Polymorphism
