# Supplementary figures and images for: Genetic control of flowering in greater yam (Dioscorea alata L.)
Source: BMC Plant Biol. 2021 Apr 1;21:163. doi: 10.1186/s12870-021-02941-7 (PMC8015048; doi:10.1186/s12870-021-02941-7)

**A)**


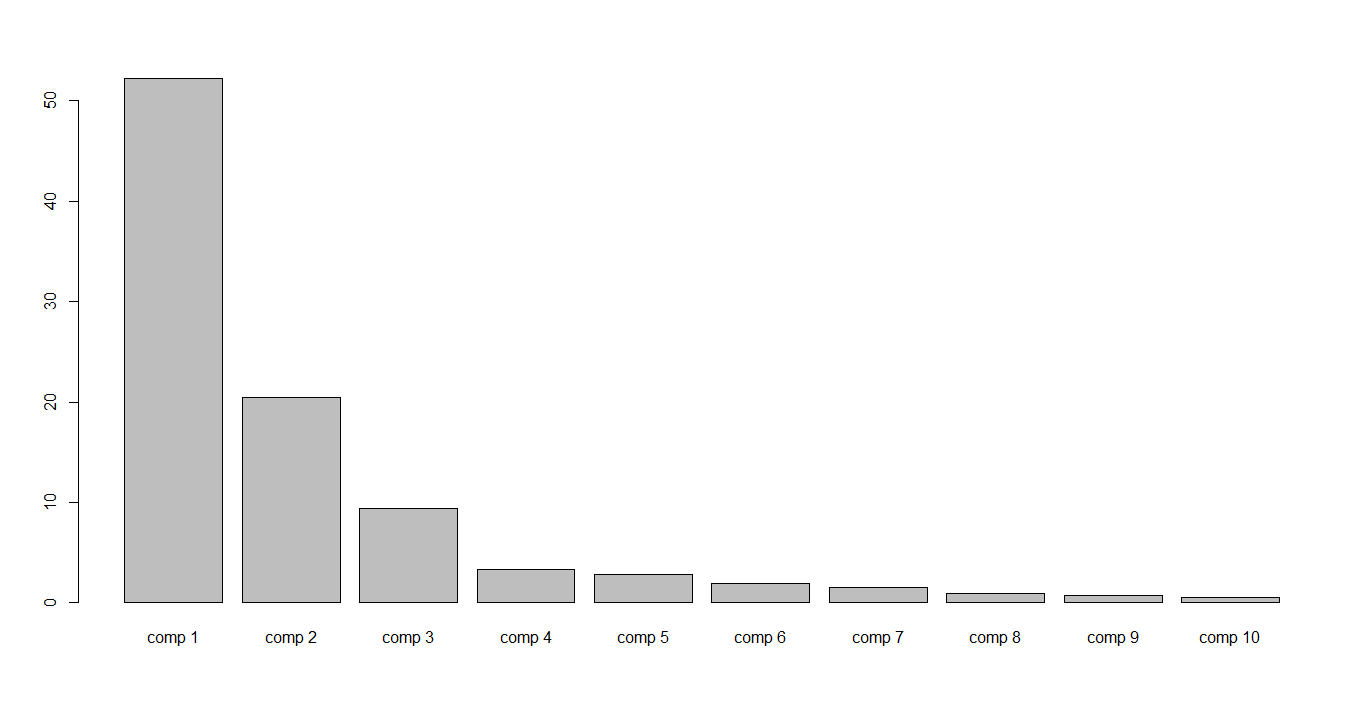


**B)**


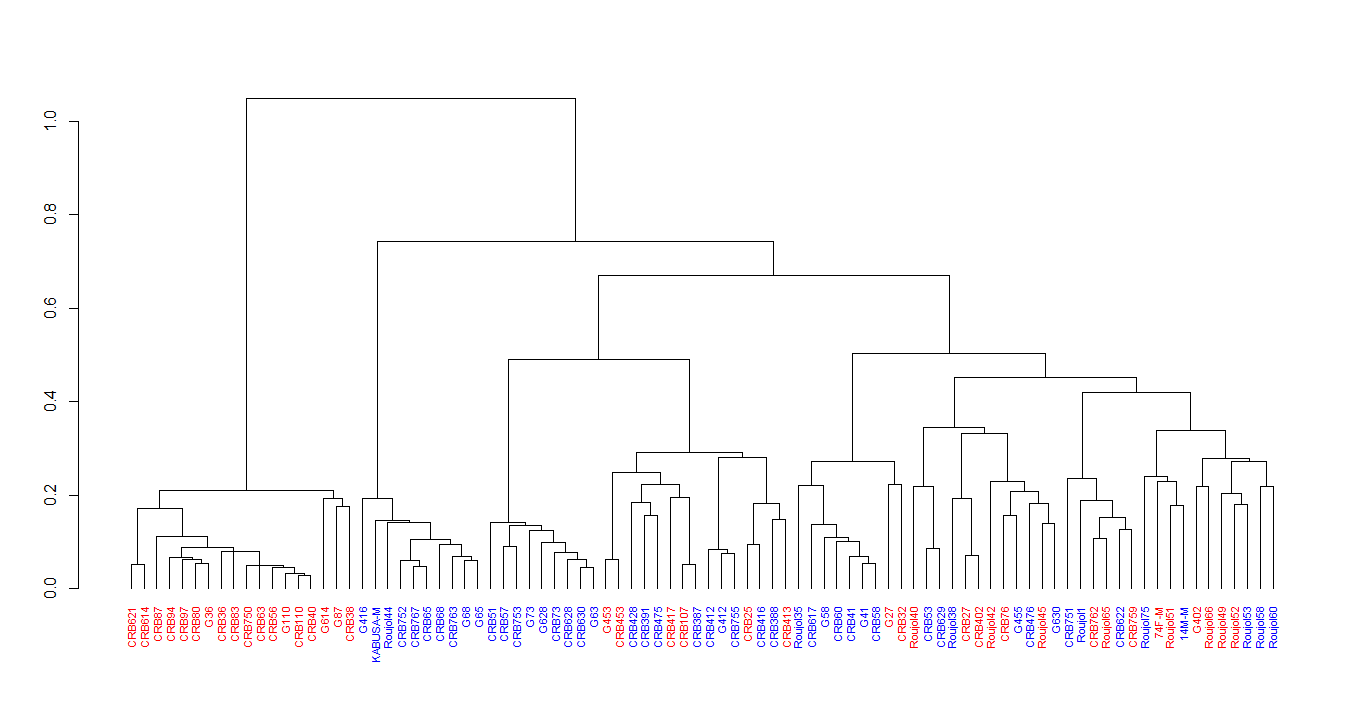


**C)**


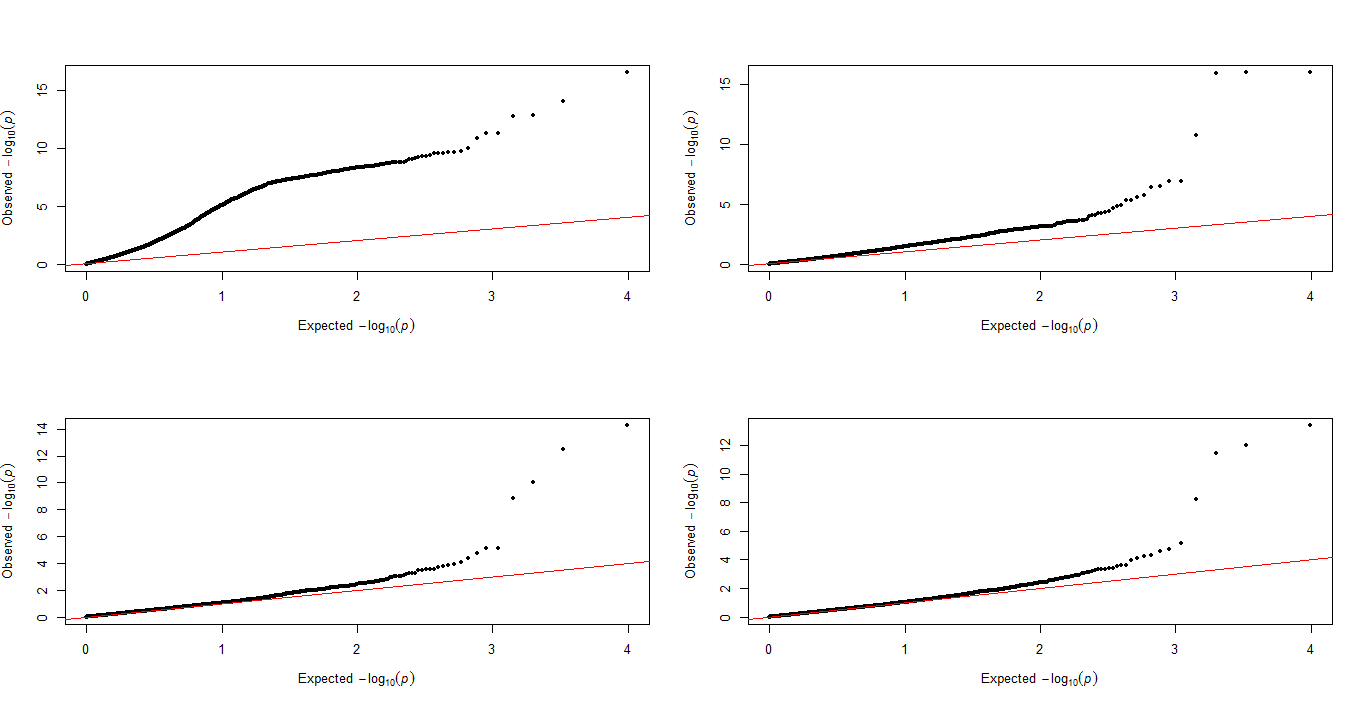

Supplement: Supplementary file 1 — Additional File 1: Fig. S1. Details on GWAS on sex determination (female or male flowering accessions). A) Barplot of the first 10 PCA eigenvalues computed to assess the panel structure. B) Clustering of accessions based on coordinates on the first five PCA axes. R cran, hclust function, “ward.D2” method. Red, female accessions; blue, male accessions. C) QQplot on GWAS results. Up/left, generalized linear model; up/right, P model; down/left, K model and down/right PK model. [file 12870_2021_2941_MOESM1_ESM.docx]

**A)**


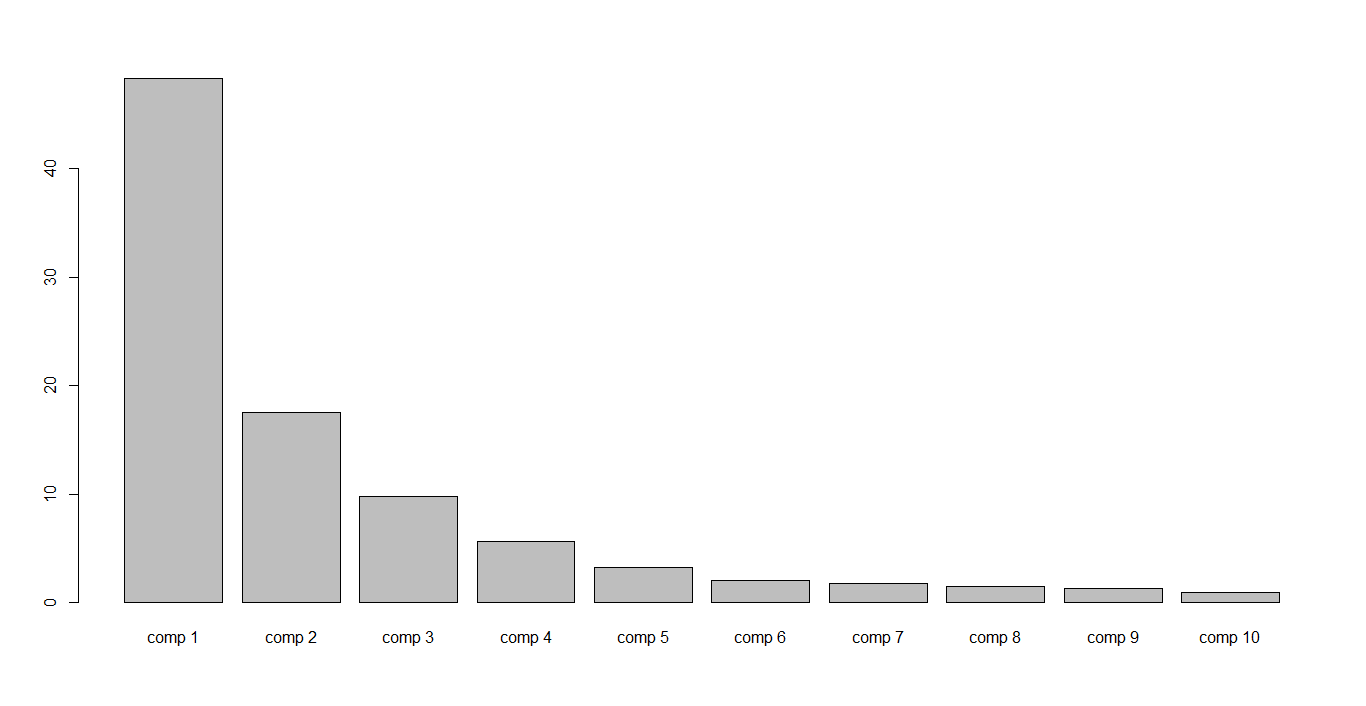


**B)**


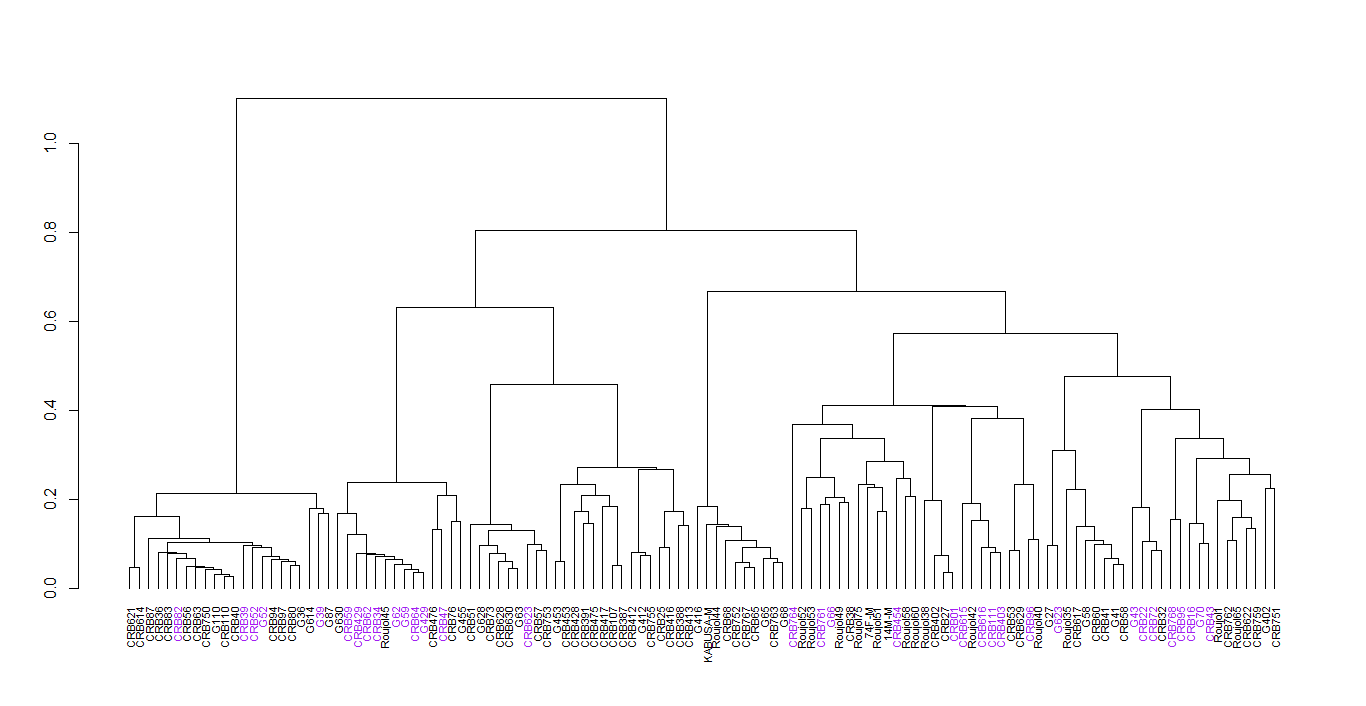


**C)**


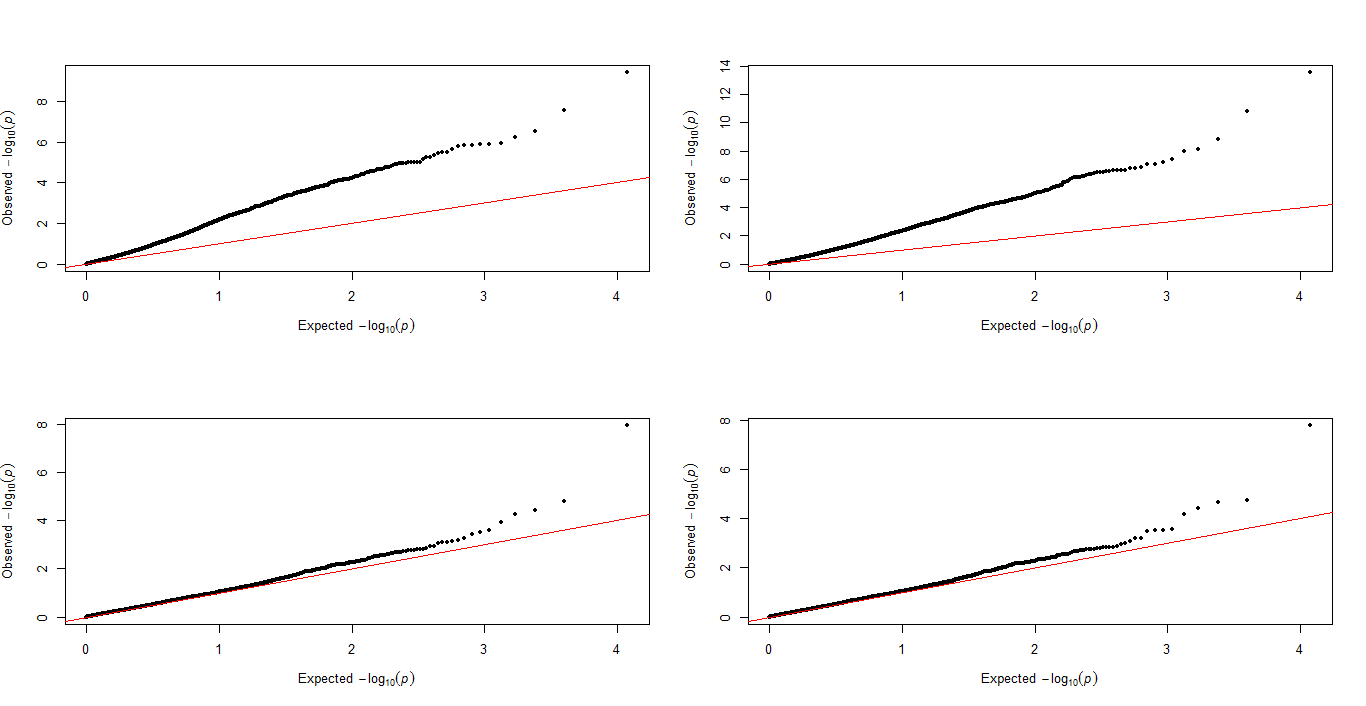

Supplement: Supplementary file 2 — Additional File 2: Fig. S2. Details on GWAS on non-flowering phenotypes. A) Barplot of the first 10 PCA eigenvalues computed to assess the panel structure. B) Clustering of accessions based on coordinates on the first five PCA axes. R cran, hclust function, “ward.D2” method. Purple, non-flowering accessions; black, flowering accessions. C) QQplot on GWAS results. Up/left, generalized linear model; up/right, P model; down/left, K model and down/right PK model. [file 12870_2021_2941_MOESM2_ESM.docx]

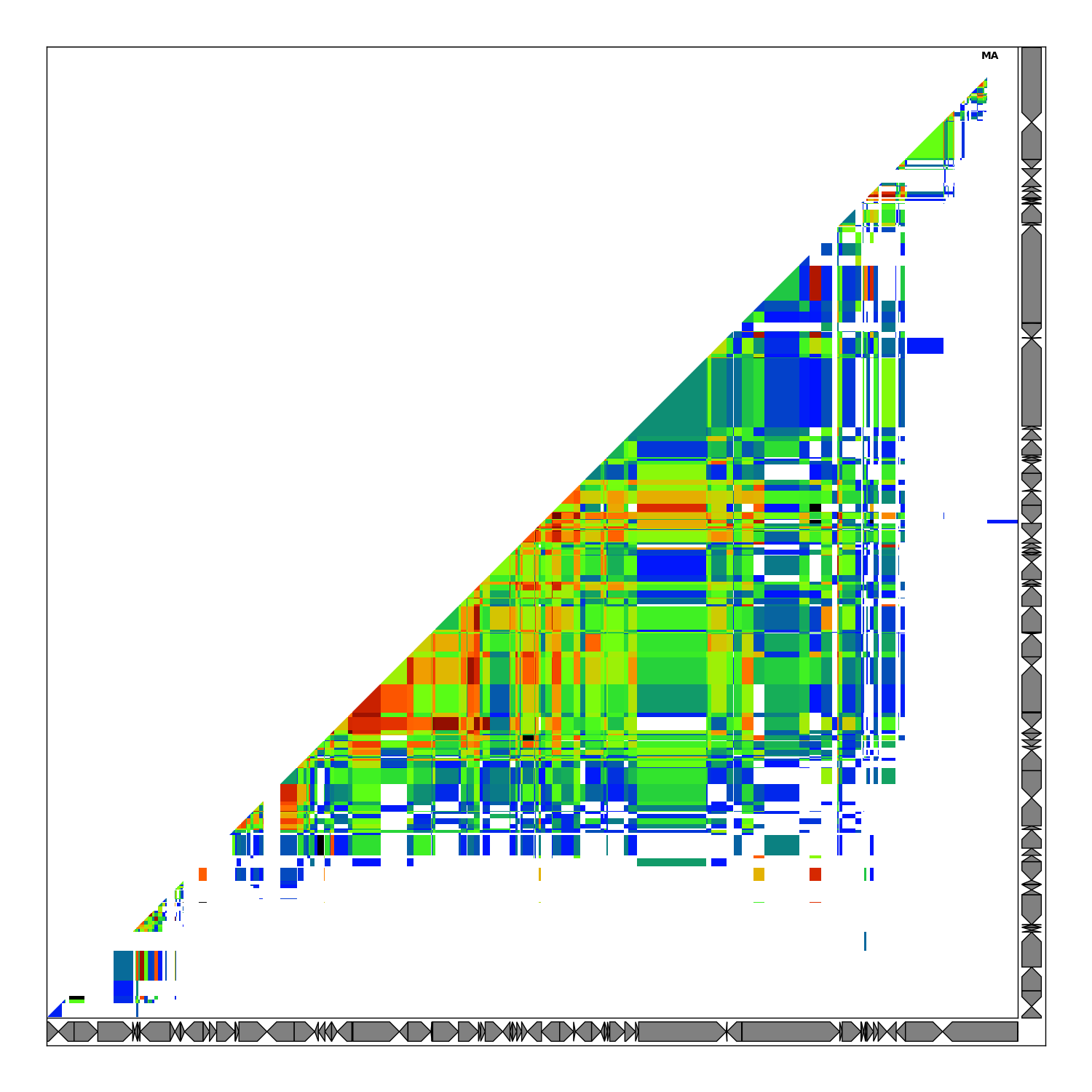


**B)**


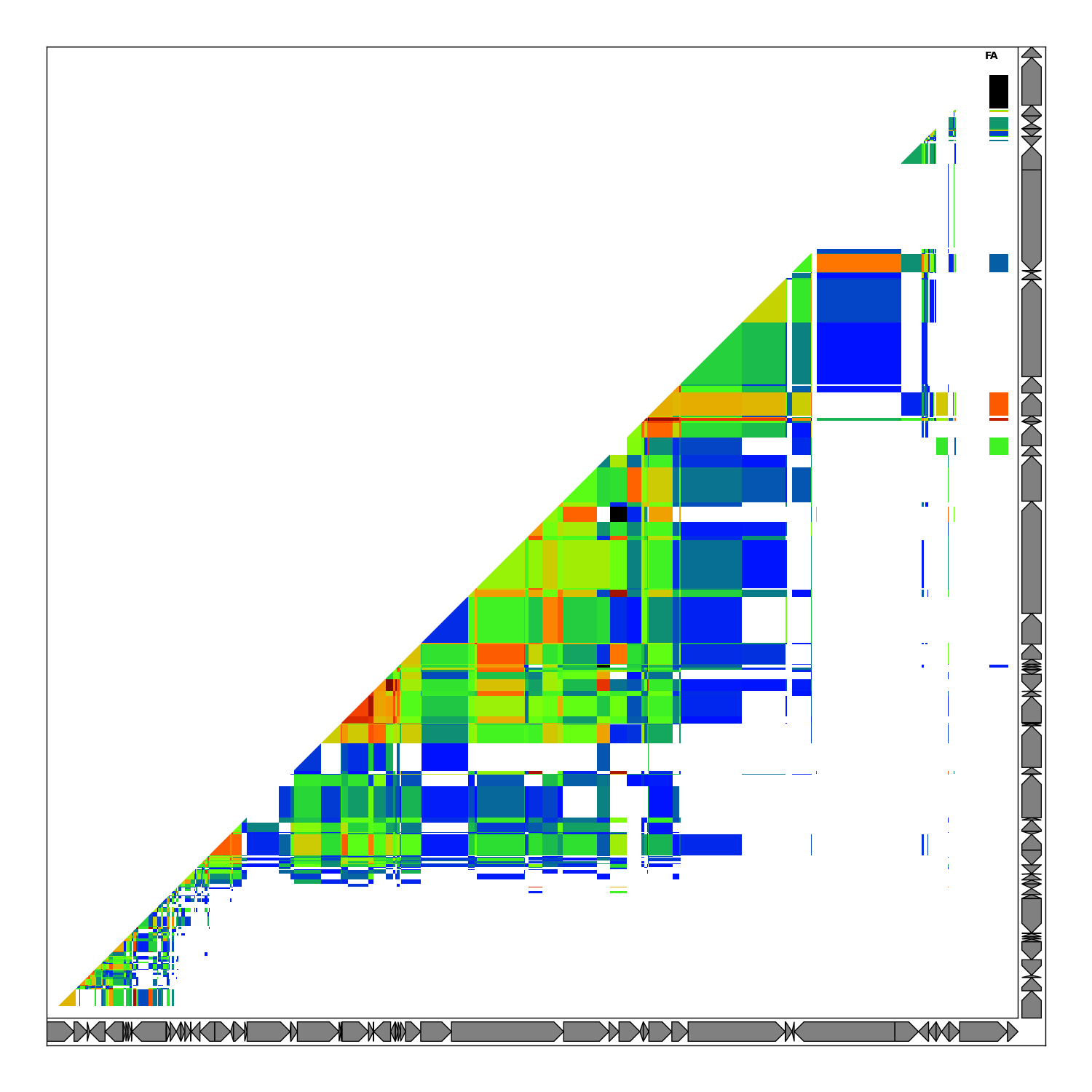

Supplement: Supplementary file 5 — Additional File 5: Fig. S5.: Dotplot of recombination frequencies along the reconstructed male and female chromosome 6 of D. alata. A) Male chromosome 6 and B) Female chromosome 6. Recombination frequencies were computed from a biparental population (74F x Kabusa) consisting of 110 progenies. Scaffolds from the D. alata genome V1 were used. Chromosome reconstruction pipeline available at: http://galaxy.southgreen.fr/galaxy/u/droc/p/scaffhunter%2D%2Dchromosome-reconstruction. [file 12870_2021_2941_MOESM5_ESM.docx]

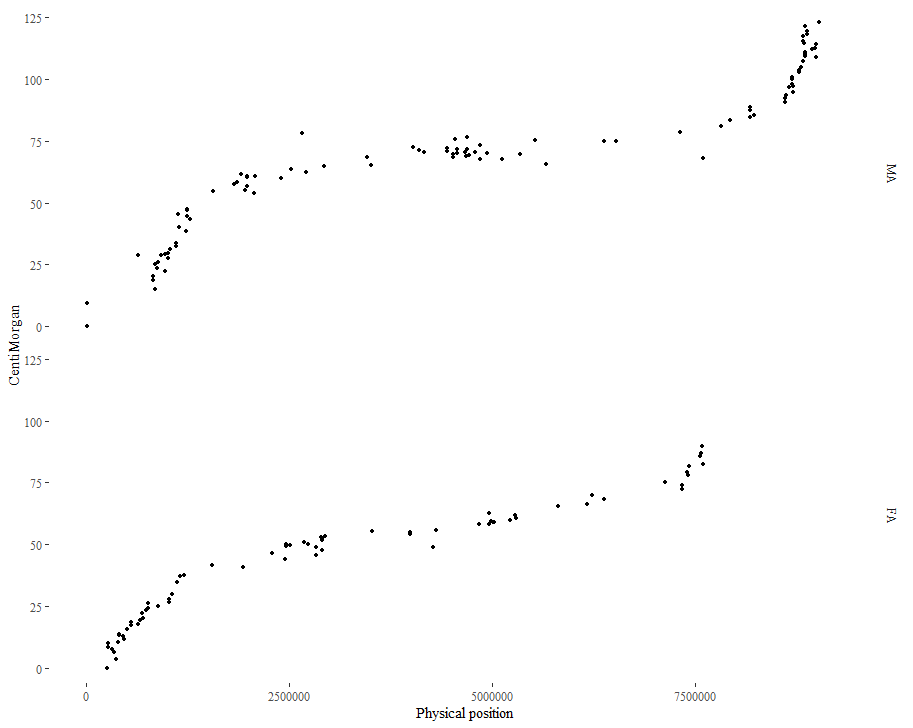

Supplement: Supplementary file 6 — Additional File 6: Fig. S6. Physical versus genetic distance along the two reconstructed sex-chromosome 6. Up, male chromosome; down, female chromosome. Genetic distance were calculated using JoinMap 4.1 software (Van Ooijen, 2012; option: recombination frequencies below 0.45, LODs over 1.0, ripple value 1, regression mapping and Kosambi mapping function). [file 12870_2021_2941_MOESM6_ESM.docx]

**A)**


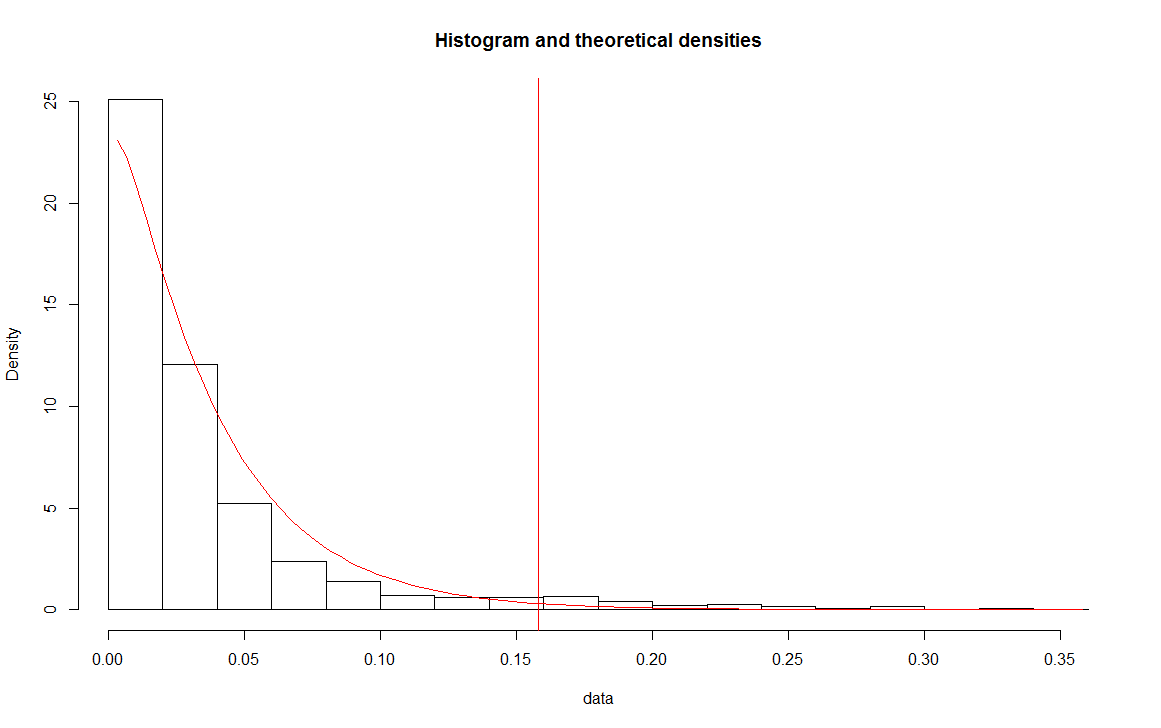


**B)**


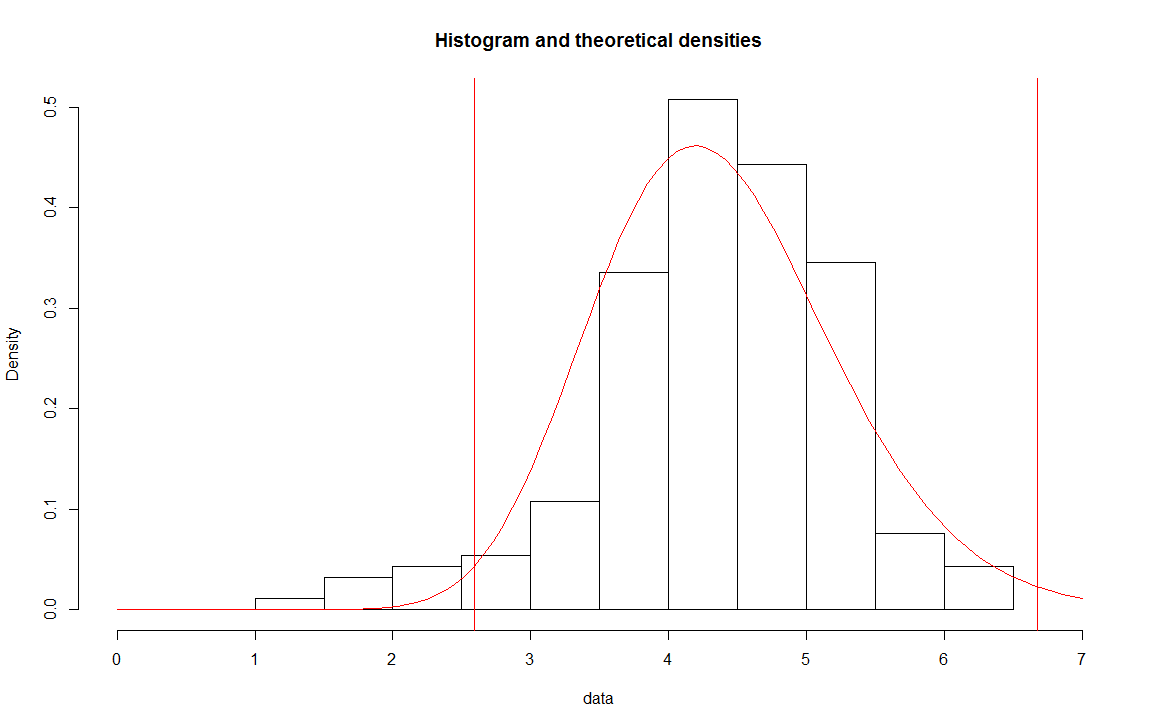

Supplement: Supplementary file 7 — Additional File 7: Fig. S7. Details on significance thresholds used in male and female resequencing comparative studies. A) Distribution of Fst between male and female and definition of significance threshold. A sliding window of 500 SNPs was used (step = 100 SNPs) to compute Fst. The significance threshold was then assessed at 0.158. B) Difference in male and female read coverage. A sliding window of 200,000 bp was used (step = 50,000 bp). Thresholds were assessed at 6.67 and 2.59 for significance of over-coverage and under-coverage for males and females, respectively. For both analyses, a gamma distribution was fitted on the empirical distribution using the fitdist function of the fitdistrplus R cran library (red curves). The significance thresholds (vertical red lines) were then assessed using a risk of 0.01. [file 12870_2021_2941_MOESM7_ESM.docx]

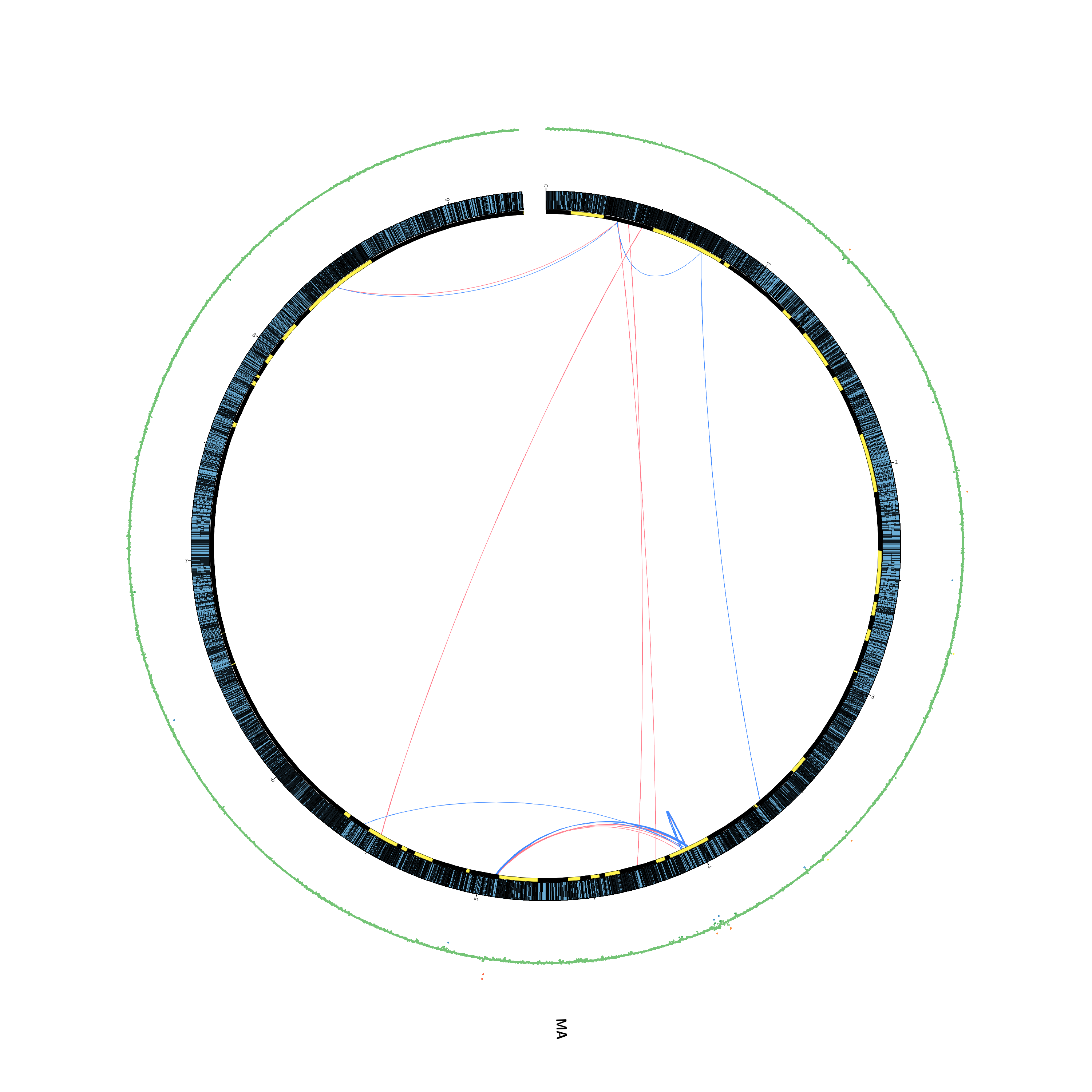

Supplement: Supplementary file 8 — Additional File 8: Fig. S8. Circos visualization of male-specific discordant read clusters. Discordant read clusters (putative structural variations) were detected using the scaffremodler package (Martin et al., 2017) available at https://github.com/SouthGreenPlatform/scaffremodler. Male and female resequencing datasets were separately used on the reconstructed male chromosome 6. Default options were used with an expected forward-reverse read orientation and a minimal and maximal insert size set at 150 and 450 bp, respectively. Then male-specific discordant read clusters were trimmed by comparing the two results files. Links color: red, deletion; blue, reverse-forward; Scaffolds, black or yellow; Barplot from black to blue depending on the male read depth and the plot of scaled coverage difference between male and female pools (step: 500 bp; window: 1000 bp). [file 12870_2021_2941_MOESM8_ESM.docx]
