# Supplementary material for: Genetic control of flowering in greater yam (Dioscorea alata L.)
Source: BMC Plant Biol. 2021 Apr 1;21:163. doi: 10.1186/s12870-021-02941-7 (PMC8015048; doi:10.1186/s12870-021-02941-7)
Supplement: Supplementary file 3 — Additional File 3: Fig. S3. Details on the genomic region linked to non-flowering/flowering phenotypes. A) Dotplot of sequencing homology between D. rotundata and D. alata. The dotplot was computed using the NCBI blastn web server (discontinuous megablast, default parameter) with the D. rotundata sequence of chromosome BDMI01000001.1 (D. rotundata genome v1 [18];) on the y-axis and the D. alata scaffold112 (D. alata genome v1) on the x-axis. B) Summary of significantly enriched G.O. terms within the genomic region. G.O. terms were extracted from the annotated D. alata transcriptome [46] mapped on the D. rotundata genome v1 and available at http://yam-genome-hub.cirad.fr/jbrowse. G.O term enrichment analysis was performed using the TopGO R cran package (“classic” Fisher test options) studying the D. rotundata genomic region from 50 kb to 250 kb on D. rotundata chromosome BDMI01000001.1. [file 12870_2021_2941_MOESM3_ESM.docx]

**A)**


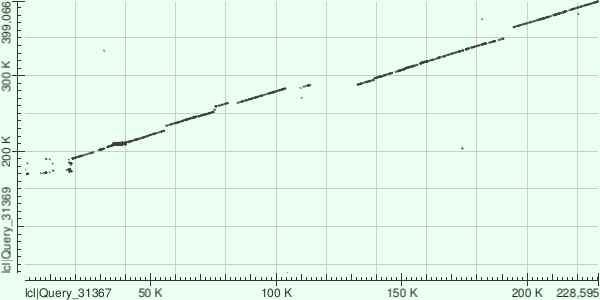


**B)**

| G.O. ID | Term | Annotated | Significant | Expected | ClassicFischer |
| --- | --- | --- | --- | --- | --- |
| GO:0048232 | male gamete generation | 50 | 2 | 0.06 | 0.0017 |
| GO:0007276 | gamete generation | 82 | 2 | 0.10 | 0.0045 |
| GO:0032504 | multicellular organism reproduction | 94 | 2 | 0.12 | 0.0058 |
| GO:0048609 | multicellular organismal reproductive process | 94 | 2 | 0.12 | 0.0058 |
| GO:0019953 | sexual reproduction | 108 | 2 | 0.14 | 0.0076 |
| GO:0044703 | multiorganism reproductive process | 108 | 2 | 0.14 | 0.0076 |
