# Supplementary material for: Genetic control of flowering in greater yam (Dioscorea alata L.)
Source: BMC Plant Biol. 2021 Apr 1;21:163. doi: 10.1186/s12870-021-02941-7 (PMC8015048; doi:10.1186/s12870-021-02941-7)

**A)**

TCTTGAAGACCATTACTTMGTGAATTAACACATGAGAAGGTTTGACACTTTCAGTTTTAGTTTAATTTAAACCTCATGATGGTTTCCTTTATGCAGCTT[A/G]CATGGATCAATGAGATCTATATTACATACTGTGGTTAGATCAGCATTAGTGTTCTGCAGTGTTCTATGGTATAATAGCATTTTGTTGGTTGCTTAATGCA

**B)**


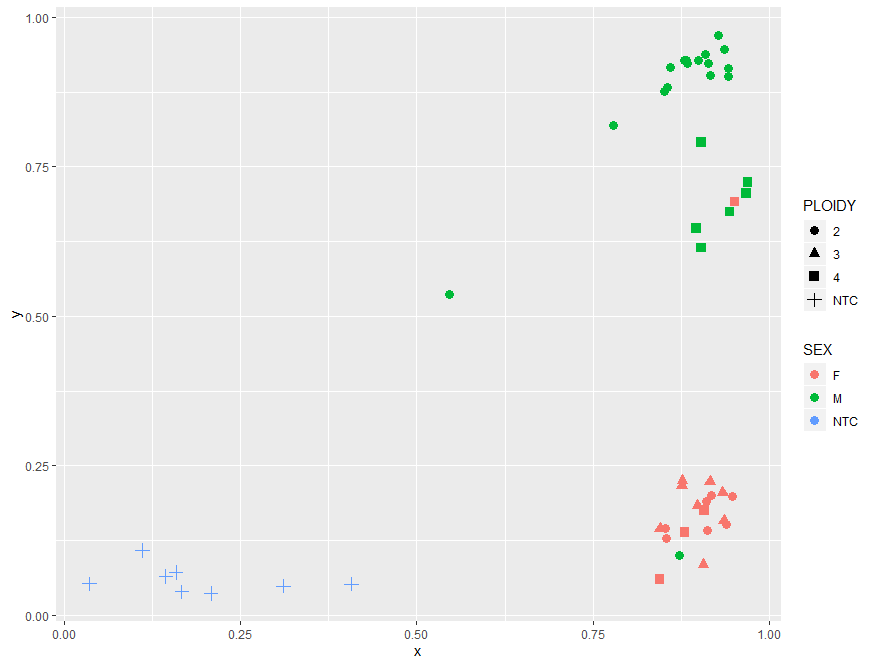

Supplement: Supplementary file 4 — Additional File 4: Fig. S4. Details on KASPar validation of the sex-related SNP (06.1_19660282). A) SNP flanking sequence and B) KASPar fluorescence results. Fluorescence signals are plotted by accession, ploidy and observed sex. In x, the “A” fluorescence allele, and in y, the “G” fluorescence allele. [file 12870_2021_2941_MOESM4_ESM.docx]
